# Alleviation of Drugs and Chemicals Toxicity: Biomedical Value of Antioxidants

**DOI:** 10.1155/2018/6276438

**Published:** 2018-12-17

**Authors:** Mohamed M. Abdel-Daim, Khaled Abo-EL-Sooud, Lotfi Aleya, Simona G. Bungǎu, Agnieszka Najda, Rohit Saluja

**Affiliations:** ^1^Pharmacology Department, Faculty of Veterinary Medicine, Suez Canal University, Ismailia 41522, Egypt; ^2^Pharmacology Department, Faculty of Veterinary Medicine, Cairo University, Giza, Egypt; ^3^Chrono-Environment Laboratory, UMR CNRS 6249, Bourgogne Franche-Comté University, F-25030 Besançon Cedex, France; ^4^Department of Pharmacy, Faculty of Medicine and Pharmacy, University of Oradea, Oradea, Romania; ^5^Department of Vegetable Crops and Medicinal Plants, University of Life Sciences, Lublin, Poland; ^6^All India Institute of Medical Sciences, Department of Biochemistry, Bhopal, Madhya Pradesh, India

Understanding the mechanism of drug- and chemical-induced toxicities is the primary interest of many researchers worldwide to develop enhanced preventive and therapeutic strategies. Paracelsus (the father of toxicology) assumed that all chemicals and drugs as well as natural products—including antioxidants—could induce toxicity when received in high doses. His rule “the dose makes the poison” is now considered the core principle of traditional toxicology. Recently, researchers understood that toxicity is a complicated process affected by many factors, including developmental exposures, genetic predisposition, and doses [1].

Antioxidants exist naturally in many beverages, fruits, and vegetables, and they can be synthesized in laboratories [2]. They delay or ameliorate cellular oxidative damage; therefore, they have several health benefits in the prevention and treatment of diseases. They could be used alone or in combination with other medications as adjuvant treatments [3].

The study of free radicals and antioxidants leads to a revolution in medicine, which includes promising new solutions in health and disease management. In the last decade, the search for effective nontoxic natural compounds with antioxidant activity has been intensified. In addition to endogenous antioxidant defense systems, the use of plant-derived antioxidants is an appropriate alternative [4, 5]. In this special issue, many original studies and review articles focused on the value of antioxidants in ameliorating drug- and chemical-induced toxicities.

To evaluate chemical-induced cytotoxicity, Radaković et al. analyzed both the biochemical and genetic effects of adrenaline toxicity in rats. They concluded that adrenaline causes nitrative and oxidative stress through which they induce damage to cellular genes, proteins, and lipids, resulting in cardiomyocyte injury. Further, Abdelazim et al. examined the oxidative effects of zinc oxide nanoparticles (ZnONPs) on the muscles of Nile tilapia, as well as the potential protective role of vitamins (C and E) via enhancement of enzymatic and nonenzymatic antioxidant systems.

Other studies investigated the benefits of antioxidants in ameliorating the toxicity of common medications with frequent toxic effects. For example, Barakat et al. investigated the protective effects of boswellic acids (BAs) against doxorubicin- (DOX-) induced hepatotoxicity in mice. They concluded that BAs modulate the hemeoxygenase-1 (HO-1) and NF-E2-related factor 2 (Nrf2) pathways, resulting in reactive oxygen species (ROS) scavenging and reduction of DNA and lipids oxidative damage. Further, Liao et al. used dl-3-n-butylphthalide (dl-NBP) to alleviate DOX-induced anxiety and depression-like behaviors through attenuation of ER stress, oxidative stress, neural apoptosis, and inflammatory reaction, providing the basis for potential preventive and therapeutic strategy against DOX-induced neurotoxicity.

The value of natural antioxidant compounds has been shown in several studies in this issue. For example, Saleh et al. concluded that berberine-rich fraction (BF) improved the infertility induced by gossypol acetate through anti-inflammatory and antioxidant mechanisms. Further, Duan et al. demonstrates that cadmium induces toxicity in the grass carp (*Ctenopharyngodon idellus)* liver through oxidative damage and activation of the caspase signaling cascade. However, treatment with Vitamin E and metallothionein alleviated cadmium hepatotoxicity through their antioxidant and antiapoptotic effects. Similarly, Palacios et al. used ascorbate to overcome the cardiovascular complications of the naphthoquinone derivative 2-(4-Hydroxyphenyl) amino-1,4-naphthoquinone in rats through inhibition of oxidative stress and reduction of blood pressure.

In addition, Abdel-Rahman and colleagues examined the antioxidant and hepatoprotective activities of the phytochemical (lycopene) against the endocrine disruptor xenoestrogen (Bisphenol A) through improving the liver function and oxidant-antioxidant balance and reducing DNA damage. In the same vein, Lebda et al. established the mechanism of miswak, *Salvadora persica*, extract ameliorative effects against ethanol-induced gastric ulcer in rats through the upregulation of transforming growth factor-*β*1 (TGF-*β*1) and endothelial nitric oxide synthase (eNOS) gene expression, improving the oxidant/antioxidant balance and mitigating the production of proinflammatory cytokines and apoptosis.

Other studies examined the value of synthetic antioxidants. For example, Guo et al. synthesized a novel dithiocarbamate, DpdtbA (di-2-pyridylhydrazone dithiocarbamate butyric acid ester), and investigated its anticancer activities. They found that it inhibits cellular growth through ROS formation and evoking p53, leading to the alteration of gene expressions related to cell survival. Further, Aly et al. used a molecular intersimple sequence repeat (ISSR) assay and cytogenetic biomarker analysis to examine the cyclophosphamide- (CP-) induced cytotoxicity and mutagenicity, as well as the potential preventive effects of the fullerene C_60_ nanoparticle (C_60_) and virgin olive oil in rats.

In this issue, a review article by Wen and colleagues summarized the physiological and pathological roles of hydrogen sulfide (H_2_S) in processes like atherosclerosis, hypertension, myocardial infarcts, and angiogenesis and examined its value as a target for drug development.
